# Seasonal changes in dry matter yield from Karst pastures as influenced by morphoclimatic features

**DOI:** 10.1371/journal.pone.0204092

**Published:** 2018-09-27

**Authors:** Cristina Pornaro, Valentina Vincenzi, Stefano Furin, Massimiliano Fazzini, Luca Minarelli, Stefano Macolino

**Affiliations:** 1 Department of Agronomy Food Natural Resources Animal and Environment, University of Padova, Legnaro, Padova, Italy; 2 Geotema Srl, Ferrara, Italy; 3 Dolomiti Project Srl, Feltre, Belluno, Italy; 4 Department of Physics and Earth Sciences, University of Ferrara, Ferrara, Italy; Wuhan University, CHINA

## Abstract

Pastures are strongly affected by local environmental variables in terms of their species richness, plant composition and herbage production. A multi-site monitoring study was conducted over three years to investigate the influence of morphoclimatic factors on the seasonal variations in dry matter (DM) yield from Karst pastures. Seven sites located on the Italian and Slovenian Karst regions were investigated that differed in terms of their geological and geomorphological features, as well as their soil types. At each site, the daily DM yield (kg ha^-1^ d^-1^) was determined using Corral-Fenlon method which permits to simulate herbage utilization from grazing herds. The morphoclimatic features were also analysed, with the aim to evaluate the link between seasonal DM yield and geomorphological and environmental factors. Generalized non-linear mixed models were built to study the observed seasonal variations in DM yield, using day of the year (DOY), growing degree days (GDD), and cumulative rainfall. Furthermore, environmental descriptors were included in the model in order to evaluate their effects on DM yield. The seasonal variations in yield showed two growing periods (spring and late summer), which were described by Gaussian curves. For the spring growing period, the model improved when the interaction between soil granulometry and growing degree days corresponding to the curve peak was taken into account. This confirms the influence of soil type and air temperature on pasture yield. For the late summer growing period, the interaction between the sand classes and the number of rainy days from the beginning of the period to the peak of the curve improved the model. The curve parameters of our models are correlated with environmental descriptors depending on the lithology and particle size of soils. The results are essential for the optimization of pasture management and avoiding degradation due to over- or under-grazing.

## Introduction

Karst grasslands, which grow on shallow soils with basic, neutral or slightly acidic pH values and high permeabilities, fall into the category of dry macro-mesothermal grasslands developed on neutral or alkaline soils [1]. According to other authors, environmental factors, especially temperature and the characteristics of the substrate, strongly affect the species richness, plant composition and production of pastures [1,2]. These factors influence the availability of environmental resources, such as water and soil nutrients, and control the competition among different species [3,4].

The correlations between topography, altitude or annual temperature and species richness or plant composition have been widely studied (e.g., [5,6]). However, to our knowledge, few studies have investigated the relationship between pasture yield and climate or environmental factors (i.e., [7–9]). Moreover, all these studies analysed annual production and its relationship with climatic factors at regional scales. In general, they reported positive relationships between annual precipitation and annual production, and negative relationships between mean annual temperature and annual production. Sala et al. [10] and Epstein et al. [11] introduced the importance of the interactions between soil texture and annual precipitation, which have a positive effect on the water-holding capacity of soil. Furthermore, the major experiments that have studied karst pasture yields have only reported annual values [12] and merely reemphasize their nature as a landscape type with low herbage yield. However, analysing seasonal production in pastures throughout the year and between years is a fundamental requirement to drive farm management decisions [8].

**Fig 1:**
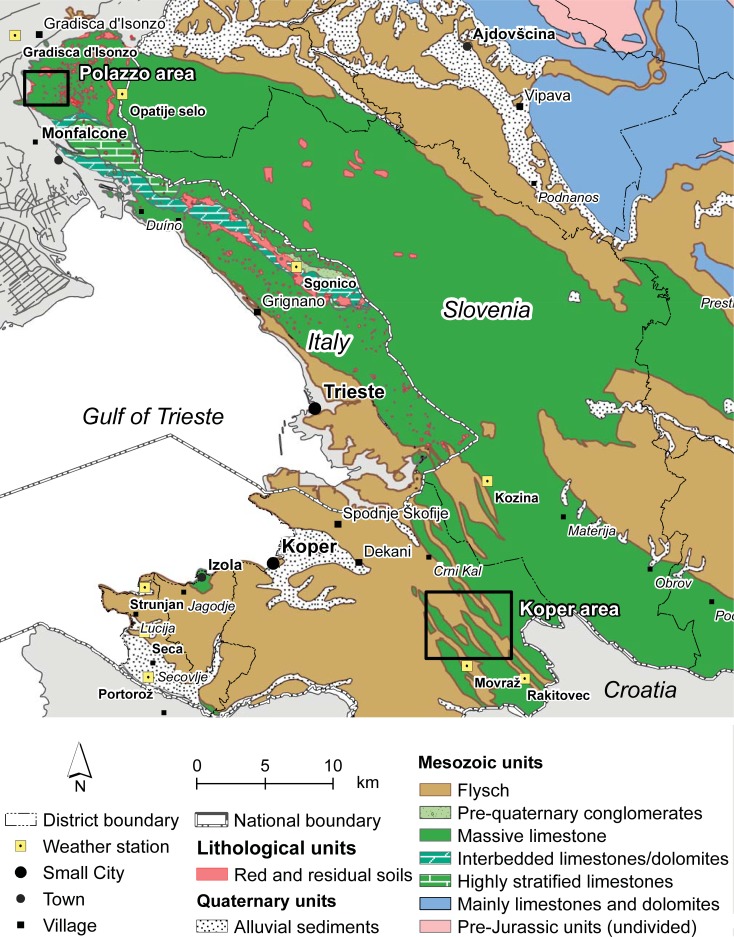
Main geographic features and lithological map of the Classical Karst; study areas and weather stations are shown on the map.

To study the seasonal DM yield of Karst pastures and the influence of environmental factors on it, an investigation was conducted for three years (2012–2014) within two study areas in the Italian and Slovenian Karst. These study areas are located in: 1) the municipality of Polazzo in Gorizia province, Italy; 2) the municipality of Koper in the Obalno-kraška region, Slovenia (Fig 1). These localities lie within the region of “Classical Karst”, a term used since the XVI century to describe the Italian and Slovenian Karsts regions as a homogeneous geological unit.

As a consequence of climate conditions and soil characteristics, Karst landscapes are known for low-intensity agriculture [13]. Pastures in this environment are also characterized by low productivity and pastoral value; consequently they face a high risk of abandonment [14] or degradation caused by overgrazing [15]. From the perspective of environmental sustainability, these pastures represent an irreplaceable resource; despite their low productivity, they support a wealth of flora and fauna [2]. For the sustainable management of these pastures, it is important to take into account seasonal variations in productivity, in order to optimally exploit their potential productivity.

The investigation aimed to verify the hypothesis that the seasonal DM yield of Karst pastures is strictly linked to geomorphology and environmental factors. Furthermore, it was hypothesized that yield changes are not only controlled by the spatial positions of the pastures, but also by seasonal and yearly climatic trends in the region. To further investigate the aforementioned hypotheses, the present study was intended to 1) fill the knowledge gap on the seasonal variations in daily Karst pasture yield; 2) analyse the influence of climate trends on yield at the study areas; 3) evaluate the link between seasonal DM yield and geomorphological and environmental factors.

## Materials and methods

Seven sites were selected in two study areas of Italian and Slovenian Karst. In each study area climate analysis were performed to determine relative humidity, air temperature, rainfall, wind direction, wind gust, wind speed and dew point. Furthermore, in each study site soil samples were collected to determine soil characteristics, and herbage was harvested weekly during the growing season to calculate daily DM yield.

### Study area

The Classical Karst is a broad area, approximately 40 km long and 15 km wide, located along the border between Italy and Slovenia, bounded by the Isonzo/Soča River to the northwest, by the Vipacco/Vipava River valley to the north and northeast, by the Pivka River watershed to the east, by the Adriatic and the Gulf of Trieste to the west and, along its southern edge, by the Dinaric Karst. The Karst plateau altitudes decrease irregularly from the southeast (where the maximum altitudes are approximately 650 m a.s.l.) to the northwest (where the average altitudes are 200–400 m a.s.l.). The climate is rather complex, as a result of a climatic transition between two different sub-domains: the Mediterranean one dominant along the coast, and the sub-continental one inland. This rapid transition results in a strong thermal and meteorological contrast among areas that are very close to each other, in relation to their proximity to areas with higher relief and their interaction with the air currents of the Mediterranean basin, which may cause intense precipitation events. The average rainfall in the Classical Karst ranges from 1000 mm/yr along the coast to approximately 1800 mm/yr in the hinterland. The region features an average winter mean temperature of approximately 3.5°C and an average summer value of approximately 19.5°C. The mean annual temperature is approximately 12°C.

Geologically, the Classical Karst belongs to the Adriatic Plate. It is characterized by the transition from a Mesozoic carbonate platform environment to a Cenozoic terrigenous setting and is dominated by the Alpine-Dinaric orogen. It represents one of the best places to observe karst geomorphology and hydrogeology. Its characteristic landscape and its karstic features are controlled by three factors, lithology, structural geology and weathering, that have acted over a large time period, from the Albian (approximately 113 Ma, [16]) to the present day. Geological features strongly control floral and faunal biodiversity, the endemic adaptation of species and habitat creation and have thus driven human activities in the area. The lithologies that outcrop in the area can be roughly divided into three main groups: Mesozoic carbonates, “Flysch” (a mixed terrigenous-carbonate turbiditic sequence), and Pleistocene-Holocene alluvial deposits [17,18]. The carbonate units are the oldest sediments, and they crop out within a wide area from the Isonzo/Soča river to the North that straddles the Italian-Slovenian boundary to the southeast and extends as far as the Rosandra Valley/Dolina Glinščice and to the northern part of the municipality of Koper. In this paper, only the geological formations outcropping at the study sites are described, based on the Italian nomenclature defined in Cucchi et al. [19] and a simplified lithological map is presented in Fig 1. The Classical Karst is characterized by an asymmetric NW–SE anticlinal structure: the strata generally dip 10–30° towards the southwest, with local increases in the inclination (up to vertical orientation) in the Duino–Mt. Grisa area. The entire area is then overlain by the main compressive structure, the Karst thrust and, in the southwestern portion of the area, by minor thrusts that repeat part of the flysch series (see also [19–22]).

The lithological and structural components of the area exert strong control on its hydrology and geomorphology, and therefore its landscape. Karst landscapes are generally characterized by 1) the absence of permanent surface flows and by the presence of swallow holes (dolines) and closed depressions; 2) the common occurrence of caves and 3) the existence of large springs, which are frequently located at the base of the carbonate sequence. As the lithology influences the susceptibility of rocks to dissolution by meteoric water, all the carbonate units are prone to the development of karst landforms and are characterized by the absence of surface hydrology but the presence of deep groundwater flows. On the other hand, the terrigenous units (flysch) represent a low-permeability medium into which rain water infiltrates more slowly, and so a surface hydrographic network can develop.

### Study sites

**Fig 2:**
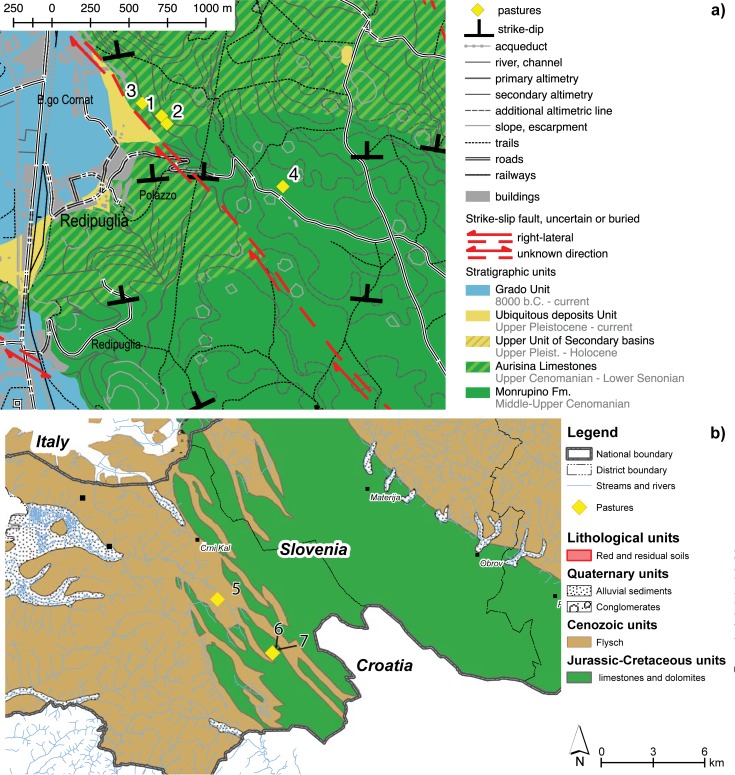
**Geological maps of a) the Polazzo area; b) the Koper area**.

Seven study sites were selected within 2 study areas, the municipality of Polazzo in Gorizia and the municipality of Koper, which are located in the Italian and the Slovenian Karst regions, respectively. These study areas are henceforth named the “Polazzo area” and the “Koper area” (Fig 1 and Fig 2). The site locations were chosen on the basis of their geomorphological and soil features. The two areas mainly differ in terms of their geological substrates, which consist of limestones in the Polazzo area and terrigenous flysch in the Koper area.

In the Polazzo area, the study sites (nr. 1, 2, 3 and 4) are located on the boundary between the Monrupino Fm. and the Aurisina Limestones Fm. (Fig 1, Fig 2A). The top of the Monrupino Fm. is characterized by compact limestones and overlaid by a transgressive, fining-upward sequence. The lower strata of the Aurisina Limestones crop out at the easternmost study sites and are markedly coarser and more porous limestones (rudstones).

The Pliocene to Holocene deposits are also represented and are dominated by “Terra Rossa” (red soil) deposits, that are clayey soils produced by the intense weathering of carbonates (the clay is the insoluble, residual sediment that was highly dispersed within the limestones). The specific climatic conditions (i.e., Mediterranean to continental temperatures plus intense rainfall) allow for measurable alteration of the aluminium and iron oxides from bauxite- to limonite- rich deposits.

In the Koper area, the study sites (nr. 5, 6 and 7) lie on the Flysch Fm. (Fig 1 and Fig 2B). The Flysch marls at the base of the turbiditic sequence show low carbonate contents and medium textural maturity. The central part of the series, which may be as thick as 500 m, contains grains that range from sand to silt in size. From the Flysch sediments, pedogenic processes generate a rich and productive soil that hosts herbaceous to woody plants.

The Polazzo area is a nearly flat plateau that lies at an elevation of approximately 100 m a.s.l. Karstic landforms, mainly dolines, are ubiquitous there. The depths of the dolines range from a few meters (in the southwest) up to 20 m (to the northeast); the average radius is approximately 50 m, and their shapes are generally regular and rounded. Dissolution features are very common at scales ranging from cm to dm, whereas larger structures are limited to areas that are crosscut by faults.

The Koper area, on the other hand, shows a geomorphology that is strictly controlled by structural geology, with a greater range of elevations that extends from 50 to 900 m a.s.l. The valleys are oriented along Dinaric structures and correspond to the footwalls of SW-verging thrusts. The lithologies in the lower part of the valley are terrigenous, and karst landforms are scarce there, whereas the northern cliffs and the top of the ridge are made up of carbonates and are more prone to develop karst landforms.

### Climate analysis

Precipitation and temperature time series were collected from all the available weather stations near the sites for the duration of the investigation; when available, historical series have also been acquired. For the Koper area, the climatological analysis was conducted on rainfall datasets that cover the period 1961–2014 collected at five stations located within a radius of approximately 20 km of the sites, specifically the Strunjan/Strugnano and Seča/Seca stations close to the coast, and, moving inland, the Movraž/Movrac, Rakitovec and Kozina stations. Temperature datasets were only available from the Portorož/Portorose Airport (close to the coast) and Sgonico/Zgonik stations (on the Karst plateau, approximately 10 km from the sea) and are limited to the period 1990–2013. For the Polazzo area, the analyses are based on the long time series from the Gradisca d'Isonzo station and have been complemented using data from the rain gauge data at Opatje Selo (in Slovenia). Table 1 lists the reference stations, which are part of the ARPA FVG Regional Meteorological Observatory (AFRMO) network on the Italian side and the National Meteorological Service of Slovenia (NMSS) network on the Slovenian side. Their locations are shown on the map in Fig 1.

**Table 1:** Weather monitoring stations used for the climatological analysis.

Station Name	Network	Latitude (°)	Longitude (°)	Altitude (m a.s.l.)
Strunjan	NMSS	45°31'55.54"	13°30'24.99"	2
Seča	NMSS	45°29'47.02"	13°36'05.28"	2
Movraž	NMSS	45°28'36.03"	13°55'04.92"	210
Rakitovec	NMSS	45°28'05.90"	13°58'09.88"	520
Kozina	NMSS	45°36'28.86"	13°56'13.28"	490
Portorož Airport	NMSS	45°29'41.59"	13°35'37.39"	2
Sgonico	AFRMO	45°44'04.33"	13°44'52.22"	268
Gradisca d'Isonzo	AFRMO	45°53'34.36"	13°30'00.24"	29
Opatije selo	NMSS	45°51'05.06"	13°34'54.03"	165
Station A	Portable station	45°29'44.02"	13°55'48.07"	400
Station B	Portable station	45°31'20.76"	13°53'22.90"	95

Two portable weather stations (WatchDog 2700 Station, Spectrum Technologies, Inc., Plainfield, IL), which were standard WMO meteorological stations, were expressly installed in the Koper area; they were precisely located in pastures n. 5 and 6 (Station A) and pasture n. 7 (Station B) at altitudes of 400 m and 95 m a.s.l., respectively. The relative humidity (%), air temperature (°C), rainfall (mm), wind direction (°), wind gust (km/h), wind speed (km/h) and dew point (°C) were measured hourly from 01/01/12 to 31/12/14.

Daily data were derived from the hourly data by means of sums (rainfall) or averages (relative humidity, air temperature, and wind speed). The cumulative rainfall was calculated from the daily rainfall data, starting from the 1^st^ of January for each year. The growing degree days (GDD) were calculated from January 1^st^ by taking the average of the daily maximum and minimum temperatures compared to a base temperature of 4°C, according to Hutchinson et al. [23].

### Soil characterization

Soil samples were collected from the different study sites every year at the beginning of the experiment. The soil samples were analysed for the following parameters: soil pH (1:5 soil: water solution), N content (total nitrogen; [g kg^-1^]), available P content (Olsen method; [mg kg^-1^]), exchangeable K content (barium chloride method; [mg kg^-1^]), and soil organic matter (Walkley and Black method; [g kg-^1^]). C:N ratios were also measured, according to Italian standard soil analysis techniques [24]; furthermore, the soil samples granulometric textures were evaluated according to G.U. [24] and the acquired data were plotted into USDA Soil texture classification diagrams for comparison. Soil depth measurements have been attempted by striking an iron rod with a hammer until the rock was reached.

### Measurement of pasture yields

During the period from March to October in 2012, 2013 and 2014 and at each study site, herds were excluded from part of the grazing surface by means of fences. In the excluded surface, four plots (4 m^2^) replicated in two blocks were established and harvested in regular rotation once a week to determine daily dry matter (DM) yields (kg DM ha^-1^ d^-1^) using the Corral-Fenlon method [25]. With this method each plot was harvested every four weeks. At each harvest, the entire plot herbage was collected in the field and subsequently oven-dried at 65°C for 36 h and weighed to determine DM yield. The herbage daily growth was then obtained according to the method which assumes that the daily growth (*Y*) is calculated with the formula (1)

$$Y_{t}=\frac{\frac{1}{4}x_{t}+\frac{1}{4}x_{t+1}+\frac{1}{4}x_{t+2}+\frac{1}{4}x_{t+3}}{28}$$ (1)

where *x* is the yield at the end of the weeks *t*, *t*+1, *t*+2, *t*+3 [25]. The observed trend in daily DM yield has been described by Cavallero et al. [26] as typical of Mediterranean pastures and is characterized by two growing periods which extend from March to mid-June (the spring period) and from mid-August to early October (the late summer period). Gaussian models Eq (2) were used to fit the observed daily DM yields of the two periods separately to obtain DM yield growth curves for each site.

f(x)=a*e−(x−b)2c2 (2)

where f_(*x*)_ is daily DM yield and *x* is day of the year (DOY).

The curve parameters *a*, *b* and *c* have biological interpretations and were used to describe the fitted curves. *a* is the peak of the curve, *b* is the DOY in which the maximum DM yield occurred, and *c* is the curve amplitude. Total DM yield (TY) produced throughout the two growing periods was estimated for every year at each site by calculating the integral of its daily growth curve. The number of days necessary to reach 90% of the DM yield (ND90) was also calculated.

### Statistical analysis

Generalized non-linear mixed models (GNLMMs) were built for both the spring and late summer periods in order to study the observed variations in seasonal DM yield as affected by DOY, GDD, and cumulative rainfall.

The analysis started with the simplest model analysing the relationship between daily DM yield and DOY or GDD, in which all parameters were independent of environment descriptors. The selected evaluated environmental descriptors were growing degree days corresponding to curve parameter *b* (GDDb), cumulative rainfall corresponding to curve parameter *b*, class of sand parameter (defined by increments of 20% in sand content: SC I for sand<20%; SC II for 20%<sand<40%; SC III for 40%<sand<60%), class of clay parameter (defined by increments of 20% in clay content: I for clay<20%; II for clay>20%), soil depth, soil N content, soil P content, soil K content, soil organic matter, and C:N ratio. For the late spring period, cumulative rainfall and the number of rainy days from the summer growth break (DOY = 200) to the DOY corresponding to curve parameter *b* were also included as environmental descriptors. Therefore, model parameters were made dependent on environmental descriptors, using the single effects of the descriptors first and then their interactions. The selection of the best model was made using the Akaike information criterion (AIC). The significance of the explanatory variables was tested using the likelihood-ratio statistic (LRS).

Once the curve parameters were estimated, Sites were grouped according to the sand classes. Spearman’s correlation coefficients of curve parameters, ND90, TY, PCb, and GDDb were calculated within each group.

Statistical analyses were performed using the R software package, v. 3.1.3 (R Development Core Team).

## Results

### Dry matter yield

**Fig 3:**
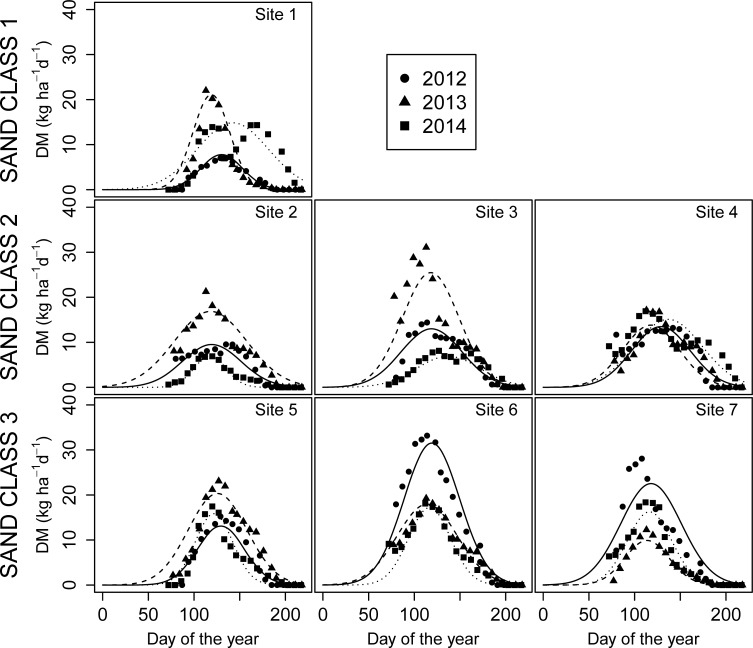
Daily dry matter (DM) yield of sites in the Polazzo area (Sites 1, 2, 3 and 4) and the Koper area (Sites 5, 6 and 7) during the spring period of each of the three studied years (2012–2014).

The TY measured during the spring period ranged from 0.58 to 2.40 t ha^-1^. At each site, differences in seasonal DM yield were observed between years (Fig 3). Site 1 displayed a very low daily DM yield in 2012; in 2013 and 2014, the TY values were similar, but the ND90 was 104 days in 2014, whereas it was only 66 days in 2013. At Sites 2 and 3, 2014 was the less productive year, although parameter *b* was similar for all years, and the value of parameter *a* was lower in 2014 when compared to those of 2012 and especially 2013 (the year with higher GDD in the growing period). Site 4 displayed similar parameter values and TY for all three investigated years. At Sites 6 and 7, 2012 was the year with higher yield, while differences between 2013 and 2014 were less clear.

**Fig 4:**
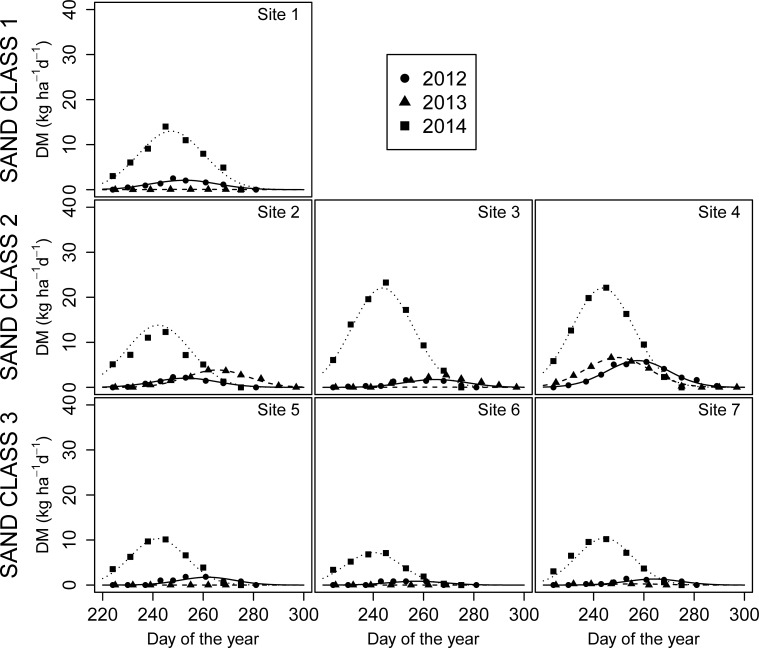
Daily dry matter (DM) yield of sites in the Polazzo area (Sites 1, 2, 3 and 4) and in the Koper area (Sites 5, 6 and 7) during the late summer period of each of the three studied years (2012–2014).

The TY of the late summer period ranged from 0 to 0.64 t ha^-1^, and it was lower than the spring TY (Fig 4). For all sites, the TY in 2014 was higher than in the other years, and the highest daily DM yield occurred earlier. For Sites 1, 5, 6, and 7, TY was zero or negligible in 2013 and very low in 2012. In contrast, Sites 2, 3, and 4 differed for TY and parameter *b*.

### Soil characterization

**Fig 5:**
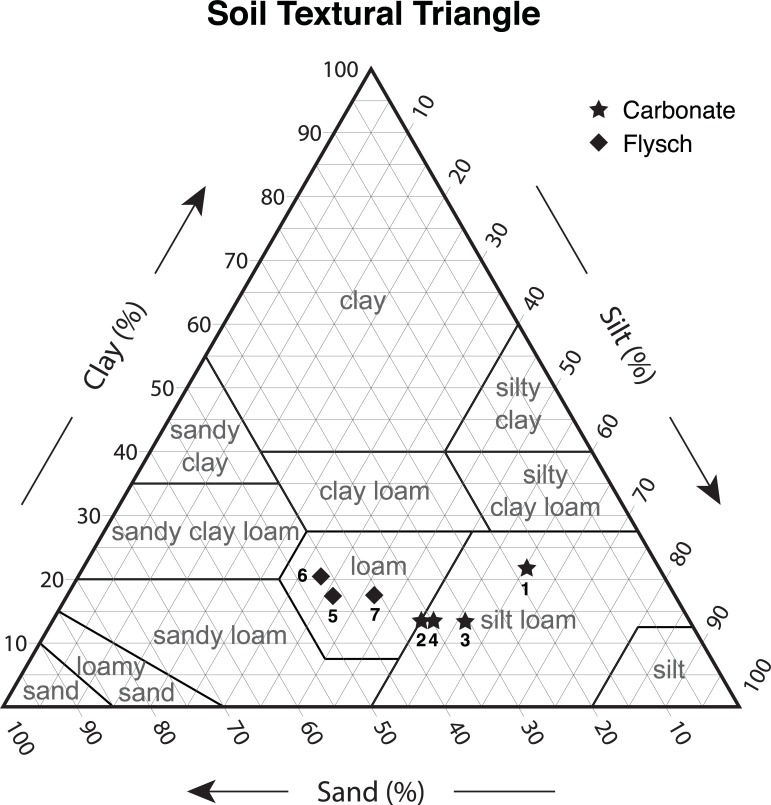
Granulometric characteristics and USDA texture classification of soil samples collected at the different study sites.

The two study areas show different particle size distributions and geochemical characteristics, both of which are strictly related to the bedrock formation. The relative abundance of coarser sediments at Sites 5, 6, and 7 are related to the original particle size of the Flysch, whereas the more abundant clay in the Polazzo area is generated by the dissolution of limestone (Table 2 and Fig 5). The soil pH at the sites ranged between 6.4 and 6.8, with the noticeable exception of Site 5 that presents a sensitively basic environment. The N and K abundances allow identification of two clusters that are named for the two study areas as well, since N and C are more abundant at the Polazzo sites (by an order of magnitude), whereas K is approximately three times higher in the Koper area. Potassium abundances are less closely related to the locations of the study sites, and they range from 0.6 to 1.4 in both areas (Table 2 and Fig 5).

**Table 2:** Particle size and geochemical characteristics of soils at the study sites.

Site ID	1	2	3	4	5	6	7
Study area	Polazzo	Polazzo	Polazzo	Polazzo	Koper	Koper	Koper
Latitude, WGS84	45°51’32”N	45°51’30”N	45°51’33”N	45°51’31”N	45°29’44”N	45°29’40”N	45°31’21”N
Longitude, WGS84	13°30’04”E	13°30’04”E	13°29’58”E	13°30’45”E	13°55’48”E	13°55’50”E	13°53’23”E
Altitude (m a.s.l.)	75	56	72	111	418	412	97
Bedrock lithology	limestone	limestone	limestone	limestone	flysch	flysch	flysch
Reference meteorologic station	Gradisca d’Isonzo	Gradisca d’Isonzo	Gradisca d’Isonzo	Gradisca d’Isonzo	Movrac Station A	Movrac Station A	Movrac Station B
Soil depth (cm)	24	14	24	13	14	17	13
Sand (%)	18.1	36.1	30.1	36.1	46.1	46.1	40.1
Silt (%)	60.0	50.0	56.0	50.0	34.0	36.0	44.0
Clay (%)	21.9	13.9	13.9	13.9	19.9	17.9	15.9
USDA soil class	silt loam	silt loam	silt loam	silt loam	loam	loam	loam
pH	6.8	6.4	6.8	6.9	8.0	6.7	6.8
N content (g kg^-1^)	2.4	3.2	4.1	3.3	0.45	0.47	0.62
C:N (g/100 g)	12.2	17.1	13.5	18.0	15.3	15.0	12.8
P content (mg kg^-1^)	1.4	0.8	0.6	1.0	0.6	0.6	1.2
K content (mg kg^-1^)	149.3	260.3	154.2	279.9	582.8	667.0	536.8

The anomalous pH value observed at Site 5 may be explained by the presence of less altered sediments that are still able to release cations into water. Nitrogen and C abundances are more likely to be explained through different degrees of alteration of the organic fraction, which is eventually related to the porosity of the soil; in more permeable soils, the combined action of water and the microfauna allow for more rapid depletion of these elements. The K abundance data reflect divergent behaviour and may be related to the composition of the substrate. The Flysch sediments are, in fact, rich in quartz, calcite, plagioclase, micas (biotite and muscovite), illite and chlorite, of which the feldspar and the phyllosilicates are potential sources of K by partial hydrolysis processes.

### Climate features

**Fig 6:**
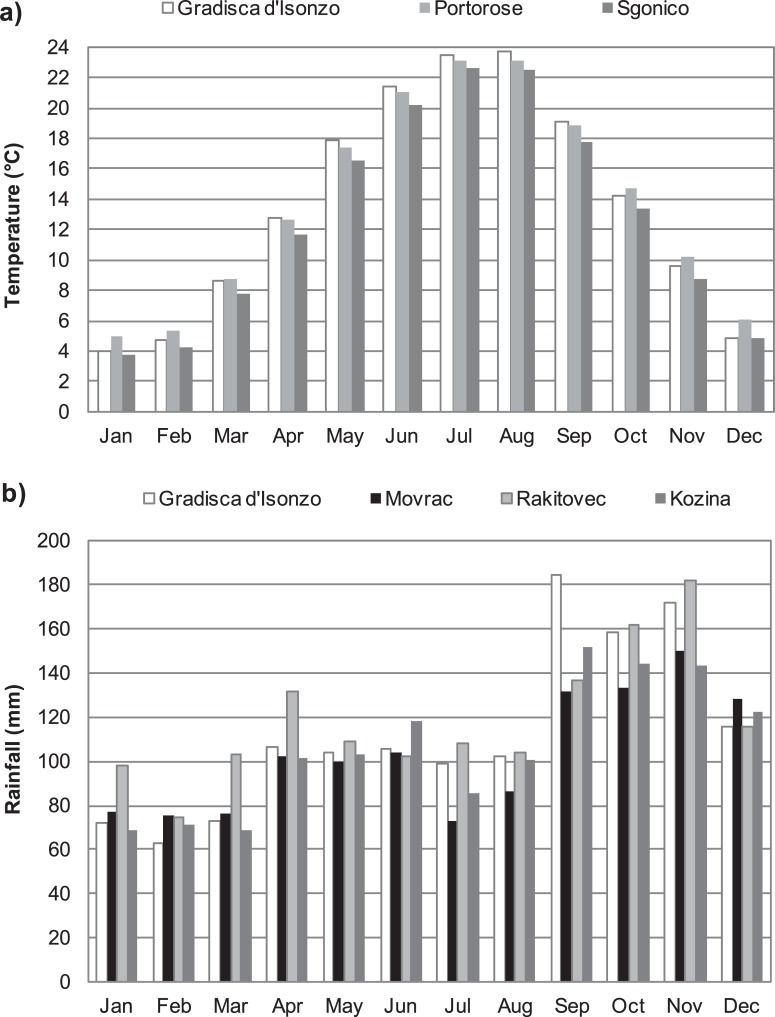
**Climate trends of the 1990–2013 average year**: a) mean monthly temperatures at Gradisca d’Isonzo (Polazzo area), Portorose, and Sgonico (Koper area); b) mean monthly rainfalls at Gradisca d’Isonzo (Polazzo area), Movrac, Rakitovec, and Kozina (Koper area).

Analysing the recent historical series from 1990 to 2013, the annual average temperature in the Polazzo area is approximately 13.7°C; the mean temperatures in January and July are approximately 4°C and 24°C, respectively (Fig 6A; Gradisca d’Isonzo station). The recent trend shows important rises in temperature of approximately 1.4°C in the last two decades. According to the Köppen-Geiger climate classification, the climate is humid temperate sub-littoral with a very hot summer (the *“Cfa”* climate sub-group). The average annual rainfall is approximately 1350 mm, distributed over approximately 98 days. The rainfall regime in this area is a sub-littoral type. The rainiest season is autumn, while the summer and spring receive almost the same cumulative total rainfall (301 mm vs. 300 mm) (Fig 6B). The trend for the last 24 years shows a decrease of approximately 3 mm/yr; however, referring to the last 50 years, the record reflects an increase of approximately 4 mm/yr.

In the Koper area the annual average temperature is approximately 13.5°C; the mean temperatures for January and July are, respectively, approximately 3°C and 24°C (Fig 6A; Portorose and Sgonico stations). The recent trend (1990–2013) is characterized by an increase in average temperature of approximately 0.6°C, slightly higher for the minimum temperatures. According to the Köppen-Geiger climate classification, the climate is temperate sub-littoral, with moderately wet to hot summers (it belongs to the “*Cfa*” or “*Cfb*” over 400 m a.s.l. climate subgroup). Annual precipitation is closely related to orography and to distance from the coast; the total average annual rainfall is approximately 1300 mm, and this amount is distributed over approximately 94 days. The rainfall regime is bimodal—"sub-littoral", with a main peak in October and a secondary one in May. The monthly minimum values were recorded in February and July (Fig 6B; Movrac, Rakitovec and Kozina stations). The half-century precipitation trends show a total decrease of 250 mm/yr, which corresponds to approximately 19% of current annual rainfall. Winter and autumn are the seasons that show the greatest decreases (approximately 1.4 mm per season). This signal is partially confirmed by the trend in rainy days, which shows a minor decline of 4 rainy days lost over the course of the last 50 years.

**Fig 7:**
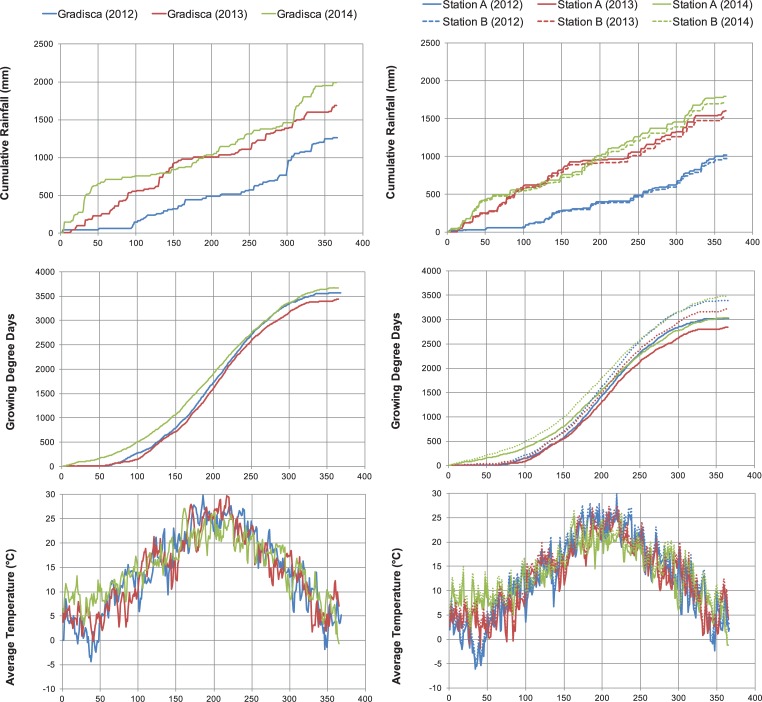
**Climate trends from 2012 to 2014 at the most representative stations**: Gradisca d’Isonzo (Polazzo area) and Stations A and B (Koper area). From the top to the bottom: cumulative rainfall (mm), GDD4 and average daily temperature (°C).

In Table 3, a synthesis of the most significant annual data collected during the 3 years of investigation is reported. It must be noted that the first monitoring year (2012) was substantially drier with respect to an average year (1990–2013); the total rainfall shows discrepancies ranging from -7 to -22%, whereas the average temperatures are slightly higher than the average year, with discrepancies of +4 to +5%. The other two monitoring years, show total rainfalls that are consistently higher than the average year and mean temperatures that are above the average values. In 2013, the discrepancies in total rainfall ranged from +23 to +25%, and those of average temperature are +3 to +4%. The year 2014 was even more peculiar, with discrepancies that ranged from +40 to +47% for total rainfall and from +9 to +10% for average temperature. These differences are evident from the graphs shown in Fig 7, where the most significant daily parameters are illustrated (cumulative daily rainfall, GDD and average daily temperatures). The year 2014 displays a peculiar cumulative rainfall trend and the highest temperature trend, with the maximum discrepancies occurring during the winter.

**Table 3:** Synthesis of climate data from 2012 to 2014: Total annual rainfall and average annual temperatures are compared with 1999–2013 average annual data, and discrepancies (%) are calculated.

			**Annual Rainfall**				
	**2012**		**2013**		**2014**		**1999–2013**
	mm	Discrepancy %	mm	Discrepancy %	mm	Discrepancy %	mm
**Gradisca d'Isonzo**	1261	-7%	1686	25%	1990	47%	1350
**Movrac**	966	-22%	1520	13%	1701	26%	1237
**Sgonico**	1199	-13%	1690	23%	1926	40%	1376
**Station A**	1019	*n*.*a*.	1604	*n*.*a*.	1795	*n*.*a*.	*n*.*a*.
**Station B**	972	*n*.*a*.	1531	*n*.*a*.	1713	*n*.*a*.	*n*.*a*.
**Average Temperature**							
	**2012**		**2013**		**2014**		**1999–2013**
	°C	Discrepancy %	°C	Discrepancy %	°C	Discrepancy %	°C
**Gradisca d'Isonzo**	14.2	4%	14.2	4%	15.0	10%	13.7
**Sgonico**	13.5	5%	13.2	3%	14.0	9%	12.8
**Station A**	12.4	*n*.*a*.	12.3	*n*.*a*.	13.2	*n*.*a*.	*n*.*a*.
**Station B**	13.6	*n*.*a*.	13.6	*n*.*a*.	14.5	*n*.*a*.	*n*.*a*.

### Seasonal distribution of pasture yield and environmental descriptors

Daily DM yield showed two growing periods: spring and late summer, with an interruption in the middle of the summer season. The data were analysed separately for the two growing seasons, which occur during the spring (growth) and late summer (regrowth).

#### Spring period

Model analyses showed that the most parsimonious model was that with the seasonal variation of daily DM yield depending on DOY. Based on the AIC, model accuracy did not improve using GDD or cumulative rainfall. When the interaction between the sand classes and GDDb were included in the model as fixed effects on parameters *a*, *b* and *c*, an improvement in the model was noted. It is interesting to note that the inclusion of this interaction was significant for parameters *b* and *c*. The random effects of the model are described in Table 4. We also tested various autocorrelation structures, but none substantially improved the model fit.

**Table 4:** Parameter estimates for mixed non-linear models (spring period) describing the effects of the day of year on parameters *a*, *b* and *c* of the curves describing pasture yield (Gaussian model). Significances are based on likelihood-ratio tests. Interaction between sand classes (SC I, II and III) and growing degree days corresponding to *b* (GDDb) was included as a fixed effect on parameters *b* and *c*.

Parameter		Coefficient	Standard error of estimate	*P*	Standard deviation (site level)
*a*		15.358	1.417	0.0000	6.294
*b*	Intercept	90.325	4.366	0.0000	0.0002
	SC II	6.888	5.572	0.2171	
	SC III	15.430	5.088	0.0026	
	GDDb	0.033	0.004	0.0000	
	SC II ×GDDb	-0.012	0.005	0.0231	
	SC III ×GDDb	-0.018	0.005	0.0005	
*c*	Intercept	-10.049	6.228	0.1075	0.001
	SC II	40.036	8.400	0.0000	
	SC III	60.754	7.464	0.0000	
	GDDb	0.035	0.006	0.0000	
	SC II × GDDb	-0.015	0.008	0.0784	
	SC III × GDDb	-0.049	0.008	0.0000	

When grouped according to the sand classes, the curve parameters were differently correlated with the environmental descriptors (Table 5). For class I, a strong correlation (c = 0.99, r = 0.99, and *P*<0.05) was found between parameter *b* (the DOY in which the maximum daily DM yield occurred) and the cumulative rainfall corresponding to *b*. For SC II, parameters *a* and *b*, as well as TY, were significantly correlated with GDDb (c = -0.57, r = 0.57, *P*<0.05; c = 0.74, r = 0.55, and *P*<0.05; c = -0.73, r = 0.54, and *P*<0.05), whereas parameter *a* was correlated with TY (c = 0.94, r = 0.89, *P*<0.001). For SC III, parameter *c* and ND90 were significantly correlated with GDDb (c = -0.74, r = 0.55, and *P*<0.05; c = -0.72, r = 0.51, and *P*<0.05) and, as with SC II, parameter *a* was correlated with TY (c = 0.96, r = 0.91, and *P*<0.001).

**Table 5:** Spearman’s correlation coefficients between parameter estimates for mixed non-linear models, number of days necessary to reach 90% of total DM yield (ND90), total DM yield (TY), cumulative precipitation until the day when maximum daily DM yield occurs (PCb), growing degree days until the day when maximum daily DM yield occurs (GDDb). All models and calculated parameters are reported for the two growing periods (spring and late summer). Coefficients are reported for significant correlations only.

Parameter	Spring period							Late summer period						
SC I	(1)	(2)	(3)	(4)	(5)	(6)	(7)	(1)	(2)	(3)	(4)	(5)	(6)	(7)
(1) *a*														
(2) *b*	/							/						
(3) *c*	/	/						/	/					
(4) ND90	/	/	0.99					/	/	/				
(5) TY	/	/	/	/				0.99	/	/	/			
(6) PCb	/	0.99	/	/	/			/	/	/	/	/		
(7) GDDb	/	/	/	/	/	/		/	/	/	/	/	/	
SC II														
(1) *a*														
(2) *b*	/							-0.85						
(3) *c*	/	/						/	/					
(4) ND90	/	/	0.99					/	/	/				
(5) TY	0.94	/	/	/				0.99	-0.86		/			
(6) PCb	/	/	/	/	/			/	/	/	/	/		
(7) GDD4b	-0.57	0.74	/	/	-0.73	/		/	0.91	/	/	-0.68	/	
SC III														
(1) *a*														
(2) *b*	/							/						
(3) *c*	/	/						/	-0.76					
(4) ND90	/	/	/					/	/	/				
(5) TY	0.96	/	/	0.77				0.99	/	/	/			
(6) PCb	/	/	/	/	/			/	-0.78	/	/	/		
(7) GDD4b	/	/	-0.74	-0.72	/	/		/	/	-0.78	/	/	/	

At sites corresponding to SC III, the TY was linked to parameters *a* and ND90 (c = 0.96, r = 0.91, and *P*<0.001; c = 0.77, r = 0.59, and *P*<0.05). Furthermore, for SC I and II, parameter *c* and ND90 were highly correlated (c = 0.99, r = 0.99, and *P*<0.05; c = 0.99, r = 0.99, and *P*<0.05).

#### Late summer period

As was found for the spring period, the most parsimonious model for the summer period was the one with seasonal variations in daily DM yield depending on DOY. Again, including GDD and cumulative rainfall did not improve the model. When environmental descriptors were included in the model as fixed effects on parameters *a*, *b* and *c*, a model improvement was found with the interaction between the sand classes (SC I, II and III) and the number of rainy days from the summer growth interruption until the values of curve parameter *b* (PDsg) as a fixed effect on *a*. The random effects of the model are described in Table 6.

**Table 6:** Parameters estimated for mixed non-linear models (late summer period) describing the effects of the day of year on parameters *a*, *b* and *c* of the curves describing pasture yield (Gaussian model). Significances are based on likelihood-ratio tests. Interaction between the sand classes (SC I, II and III) and the number of rainy days from the summer growth break corresponding to curve parameter *b* (PDsg) was included as a fixed effect on parameter *a*.

Parameter		Coefficient	Standard error of estimate	*P*	Standard deviation (site level)
*a*	Intercept	6.855	5.345	0.2018	4.41
	PDsg	0.695	0.222	0.0021	
	SC II	-1.470	6.164	0.8119	
	SC III	8.603	5.931	0.1491	
	SC II × PDsg	0.117	0.053	0.0405	
	SC III × PDsg	-0.587	0.265	0.0281	
*b*		251.555	2.045	0.0000	8.19
*c*		17.733	0.196	0.0000	0.39

When grouped according to the sand classes, the curve parameters displayed significant correlations with environmental descriptors only for classes 2 and 3 (Table 5). For class 2, significant correlations were found between parameter *b* and GDDb (c = 0.91, r = 0.82, and *P*<0.001) and between TY and GDDb (c = -0.68, r = 0.46, and *P*<0.05). For this class, there were also significant correlations between parameter *b* and TY (c = -0.86, r = 0.74, and *P*<0.01) and between parameters *a* and *b* (c = -0.85, r = 0.73, and *P*<0.01). For class 3, significant correlations were found between parameter *b* and cumulative rainfall corresponding to *b* (c = -0.78, r = 0.61, and P<0.05), between parameter *c* and GDDb (c = -0.78, r = 0.61, and *P*<0.05), and between parameters *b* and *c* (c = -0.76, r = 0.58, and *P*<0.05).

Furthermore, all classes showed highly significant correlations between parameter *a* and TY (c = 0.99, r = 0.99, and *P*<0.01 for SC I; c = 0.99, r = 0.99, and *P*<0.001 for SC II; c = 0.99, r = 0.99, and *P*<0.001 for SC III).

## Discussion

### Annual dry matter yield of Karst pastures

The total annual DM yield of the studied sites was generally poor. To our knowledge, only a few studies have reported information on Karst pasture yields [12,27]. Pornaro et al. [27] demonstrated, in a study conducted in Basovizza (45.651652 N, 13.879031 E, 405 m a.s.l.; annual rainfall, approximately 1274 mm and mean annual temperature, 12.8°C), that regularly utilized pastures provided a total annual DM yield of 1 t ha^-1^ and were therefore able to support approximately 0.3 or 0.6 livestock units per ha for lactating or dry cows, respectively. In the North Adriatic Karst (Slovenia, 820 m a.s.l.) and in pastures characterized by annual rainfalls of approximately 1834 mm and a mean annual temperature of 5.6°C, Škornik et al. [12] observed a total annual DM yield that ranged from 2.5 and 3.5 t ha^-1^, depending on grazing intensity. In this study, with the exception of two sites, the total annual DM yield did not exceed 2.5 t ha^-1^.

### Influence of climatic and meteorological trends on DM yields

The existing major studies used annual above-ground net primary production to analyse the influence of climatic factors on plant growth in grasslands [8,9,11]. The main environmental factors involved are precipitation and temperature. Our findings show significant differences in annual total yield, which was calculated for each site as the sum of the total DM yield during the two periods, among the three years of experimentation. In the Slovenian study area, yield was higher in 2012 than 2013 and 2014, while the opposite occurred in the Italian area. At Site 1, the annual DM yields in 2013 and 2014 were similar. In contrast, Sites 2 and 3 showed higher productivity in 2013 compared with 2012 and 2014.

It is well known that, at a regional scale, above-ground net primary production is positively correlated with mean annual precipitation [7–10]. Relationships between mean annual temperature and annual production have also been demonstrated at a global scale by Lieth [28]. This relationship was not confirmed by other studies [7,10], perhaps because of precipitation disturbances. However, Epstein et al. [29] demonstrated that, with total annual precipitation ranging from 450 to 500 mm, annual production decreases with increasing mean annual temperature. Our results did not confirm the presence of correlations between annual DM yield and annual rainfall or mean annual temperature, probably because the aforementioned studies were conducted at a regional scale and involved different ranges of rainfall and temperature. For example, Lauenroth et al. [7] reported a precipitation range of 200–1000 mm, and Lieth [28] reported a mean annual temperature range of 3–18°C. In our study, the annual precipitation ranged between 950 and 2000 mm and the mean annual temperature ranged from to 12°C to 15°C (Table 2 and Fig 7).

The influence of soil texture on annual production has been studied by different authors [10,11,30] as a factor that controls soil water availability. Noy-Meir [30] suggested the existence of an ‘*inverse texture effect whereby coarse-textured soils support higher production than fine soils in dry climates by limiting water evaporation*’. In our study evapotranspiration from soil has not been calculated, since the widely used formulas (e.g. Penman-Monteith or Penman [31]) include parameters not available at all the weather stations used for this study. Moreover, the evapotranspiration derived from the existing simplified equations (e.g. Hargreaves, Thornthwaite and Turc [32]) which mainly depend on temperature, was also not considered as it generates a spurious correlation between GNLMMs parameters. The relationship between annual evapotranspiration and aboveground net primary production in grasslands is positive but nonlinear, with the rate of increasing net primary production decreasing at higher levels of evapotranspiration [33]. However, in the range of evapotranspiration reported for Karst (450 and 600 mm year^-1^) [34] the aboveground net primary production slightly changes raising only about 50 g m^-2^ year^-1^ [33]. Epstein et al. [11], in a study conducted in the Great Plains, suggested that the increase in evaporation with increasing temperature negatively affects the benefits of high temperatures on annual production. Sala et al. [10] and Epstein et al. [11] found a significant interaction between precipitation and soil texture on annual production. Since soil moisture increases with precipitation, the effect of soil texture on water retained for plant production appears to be most effective in areas with high annual precipitation levels [11]. The use of soil water holding capacity for improving the previous regional scale models [7,35,36] represents a preliminary approach for incorporating this spatial concept into models. The results of our analyses using GNLMMs for both the spring and late summer periods confirmed these findings, since sand class was a significant environmental driver in both models.

### Seasonal DM yield and environmental factors

Little information is available on the monitoring of seasonal variations in DM yield at the level of individual sites [9]. As pointed out by Michaud et al. [37], a small number of studies have provided information on the temporal dynamics of pasture productivity. Moreover, in the unique Karst environment, information derived from the annual DM yield provides little information compared with that obtained from the daily DM yield. Through the Corral-Fenlon method, we were able to describe the daily DM yield using parameters of the production curve. The use of Gaussian models to assess ecological response patterns has been proposed by other authors [38], and our results corroborate its suitability to compare different production curves.

As reported by Pornaro et al. [27], the seasonal variations in daily DM yield of Karst pastures showed two productive periods (spring and late summer). This seasonal growth pattern has been described by Cavallero et al. [26] as typical of Mediterranean pastures. The findings of the present study show that the spring period was more productive than the late summer period, and that there are relevant differences in seasonal productivity between sites and within each site for the three years examined in the experiment (Fig 3 and Fig 4).

The major finding of the present study was the influence of the interaction between sand classes and GDD (spring period) and the interaction between sand classes and rainy days (late summer period) on DM yield. As reported in the previous section, the influence of soil in grasslands production is thought to be related to the interaction between the soil’s water holding capacity and precipitation [10,11]. However, we found that there is no significant interaction between the sand classes and precipitation during either the spring or the summer growth period. Epstein et al. [11] indicated that the soil water holding capacity is a limiting factor for the positive effect of precipitation on DM production. This could justify the lack of interaction between precipitation and the sand classes found in this study. In fact, in the Karst area, precipitation usually occurs as high amounts of water delivered in a short period of time. However, the relationship between precipitation and soil texture can be inferred, for the late summer period, from the significant interaction between rainy days and the sand classes.

Regarding the significant interaction found between the sand classes and GDD in spring, the importance of temperature in plant phenology throughout the year has been reported by several authors [37,39,40], who emphasized the stronger effects of temperature on DM yield in spring compared with summer and autumn. According to these findings, our GNLMM analysis emphasizes the importance of temperature (expressed as GDD) in spring, but not in late summer.

It is interesting to note that the curve parameters of our models are correlated with environmental descriptors in different ways, depending on the sand class. In particular, sites located on Flysch (Sites 5, 6, and 7) display different behaviour than the others. As previously mentioned, probably because of the high precipitation levels typical of Karst environments, the cumulative rainfall corresponding to *b* was not correlated with the curve parameters for any of the sand classes. The only exception was the class with lower percentages of sand, where the cumulative rainfall corresponding to *b* was positively correlated with parameter *b* (the DOY in which the maximum daily DM yield occurred), and the class with higher percentages of sand, where cumulative rainfall was negatively correlated with parameter *a* (the peak of the curve). Conversely, as suggested by Epstein et al. [11], temperature seemed to be more important for DM yield than precipitation, probably because of its influence on evaporation. During the spring period and for SC II, GDDb was negatively correlated with parameter *a* and positively correlated with parameter *b*, indicating that higher temperatures reduced the daily DM yield and postponed the peak of the growth curve. For SC III, GDDb was negatively correlated with parameter *c*, so higher temperatures occurring in spring reduced the growing period without influencing daily DM yield or its peak. Similarly, during the late summer period and for SC II, higher temperatures delayed the peak of daily DM yield, and for SC III, higher temperatures decreased the length of the productive period. These results suggested that daily DM yield of sites in the Flysch region (Sites 5,6, and 7) were less influenced by temperatures and rainfall than sites located in the Italian Karst.

## Conclusions

The study presented here noted that the total annual DM yield of Karst pastures was generally poor, and the daily yield was characterized by the existence of two growth periods (spring and late summer). The study also revealed that the yield of Karst pastures is closely linked to local geomorphology and environmental factors. Soil type, air temperature and precipitation are the factors that primarily affect the yield of Karst pastures. The influence of the interactions we noted between sand classes and growing degree days during the spring, and between sand classes and the number of rainy days during the late summer, suggest the primary importance of the water holding capacity of the soil on Karst pasture yield. The daily DM yield of sites located in Flysch were less influenced by temperature and rainfall than sites located on carbonate substrates.

Over the long term, the results obtained from this study can represent a very useful tool for the sustainable management of Karst pastures. They can be used to optimize the utilization of pastures, in order to avoid their progressive degradation due to over- or under-grazing. The influence of temperature and rainfall patterns, and soil type on forage productivity lead to a dynamic approach of grazing management of North Adriatic Karst pastures. Grazing system and stocking rate should be mainly adjusted to weather condition in combination with soil type. Therefore, they may vary year by year, season by season and also from area to area. This approach turns in favour of long-term sustainability of Karst livestock farming system.

## Supporting information

S1 Fig Original version of the Fig 1 reported in Vincenzi V, Furin F, Fazzini M, Zagoršek T, Jugovic J. The importance of hydrogeological, geological and climatic features in the Karst landscape for the protection of water resources and biodiversity. In: Bužan E, Pallavicini A editors. Biodiversity and Conservation of Karst Ecosystems. Padova University Press, Padova; 2014. pp. 85–106.
(DOCX)

S2 Fig Original version of the Fig 2B reported in Vincenzi V, Furin F, Fazzini M, Zagoršek T, Jugovic J. The importance of hydrogeological, geological and climatic features in the Karst landscape for the protection of water resources and biodiversity. In: Bužan E, Pallavicini A editors. Biodiversity and Conservation of Karst Ecosystems. Padova University Press, Padova; 2014. pp. 85–106.
(DOCX)

S1 Data File dataset reporting for all studied sites (1–7) and for the three experimental years (2012–2014) the daily dry matter yield (daily DM yield) and the growing degree days (GDD) calculated for the referenced day of the year (DOY). (XLSX)

## Abbreviations

DM: dry matter yield
DOY: day of the year
GDD: growing degree day
TY: total yield
GNLMMs: generalized non-linear mixed models
ND90: number of days necessary to reach 90% of total yield
SC: I for sand<20%; II for 20%<sand<40%; III for 40%<sand<60%
GDDb: growing degree days to reach maximum daily yield
PCb: rainfall until the day when maximum daily yield occurs
PDsg: number of rainy days from the summer growth break corresponding to curve parameter *b*
