# Supplementary material for: Seasonal changes in dry matter yield from Karst pastures as influenced by morphoclimatic features
Source: PLoS One. 2018 Sep 27;13(9):e0204092. doi: 10.1371/journal.pone.0204092 (PMC6160016; doi:10.1371/journal.pone.0204092)
Supplement: Supplementary file 1 [file pone.0204092.s001.docx]

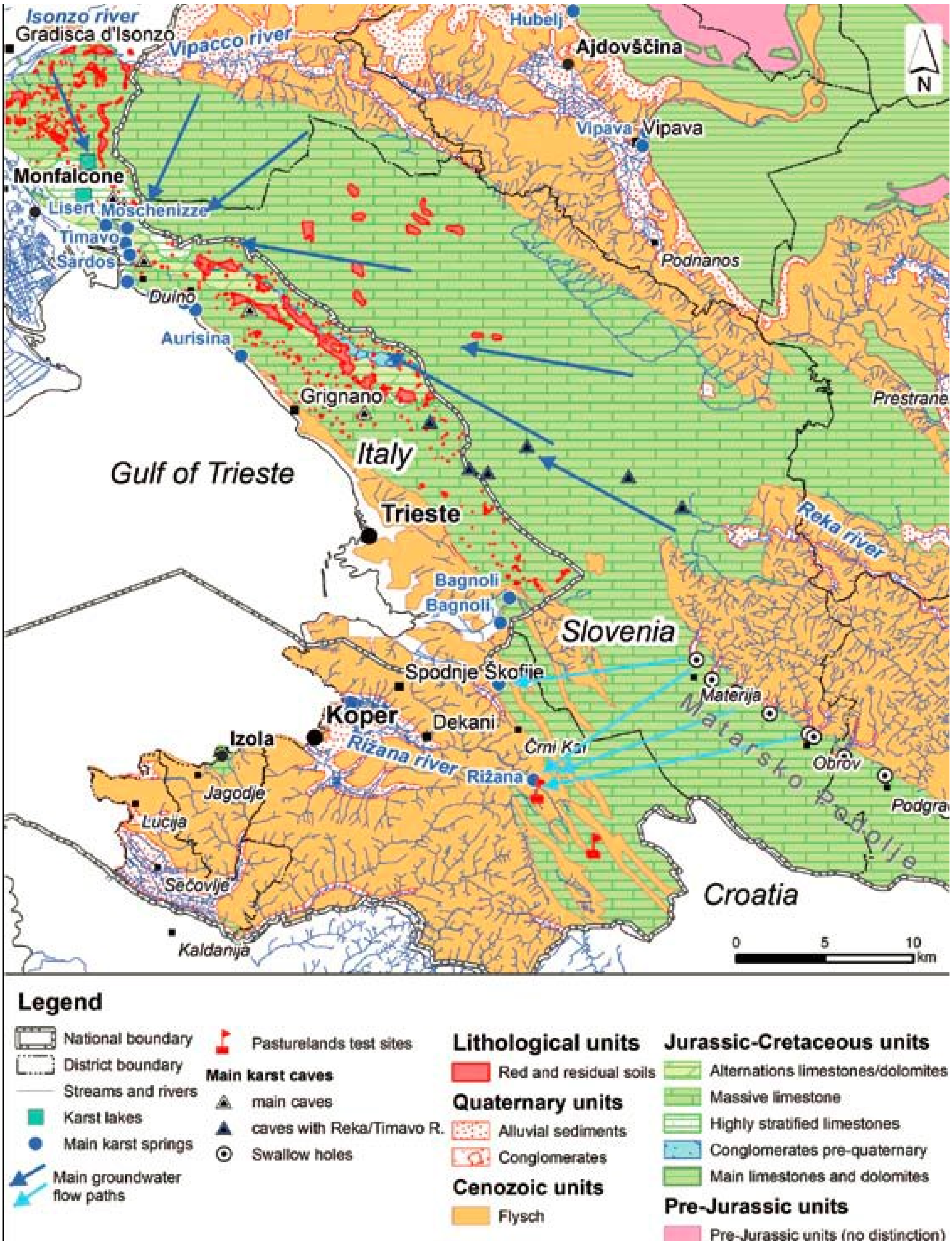
Isonzo river
Gradisca d'Isonzo
Vipacco river
Hubelj
Ajdovščina
N
Vipava
Vipava
Monfalcone
Lisert
Moschenizze
Timavo
Sardos
Duino
Podnanos
Aurisina
Grignano
Prestrane
Italy
Gulf of Trieste
Trieste
Reka river
Bagnoli
Bagnoli
Slovenia
Spodnje Škofije
Materija
Koper
Dekani
Izola
Rižana river
Črni Kal
Rižana
Obrov
Jagodje
Podgra
Matarsko Podolje
Lucija
Sečovlje
Croatia
Kaldanija
0 5 10 km
Legend
National boundary
District boundary
Streams and rivers
Karst lakes
Main karst springs
Main groundwater flow paths
Pasturelands test sites
Main karst caves
main caves
caves with Reka/Timavo R.
Swallow holes
Lithological units
Red and residual soils
Quaternary units
Alluvial sediments
Conglomerates
Cenozoic units
Flysch
Jurassic-Cretaceous units
Alternations limestones/dolomites
Massive limestone
Highly stratified limestones
Conglomerates pre-quaternary
Main limestones and dolomites
Pre-Jurassic units
Pre-Jurassic units (no distinction)
