# Supplementary material for: Seasonal changes in dry matter yield from Karst pastures as influenced by morphoclimatic features
Source: PLoS One. 2018 Sep 27;13(9):e0204092. doi: 10.1371/journal.pone.0204092 (PMC6160016; doi:10.1371/journal.pone.0204092)
Supplement: Supplementary file 2 [file pone.0204092.s002.docx]

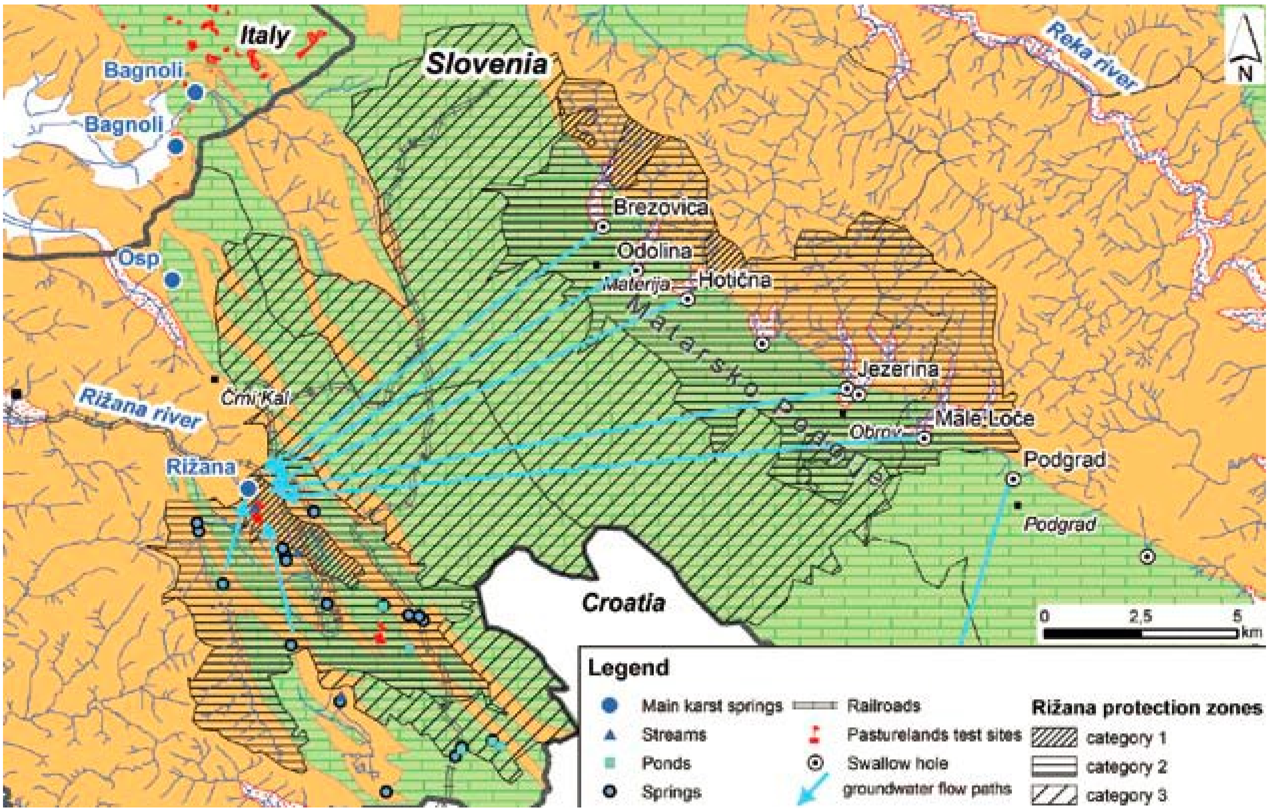
Italy
Slovenia
Croatia
Reka river
Bagnoli
Bagnoli
Osp
Rižana river
Črni Kal
Rižana
Brezovica
Odolina
Materija
Hotična
Matarsko Podolje
Jezerina
Male Loče
Obrov
Podgrad
Podgrad
N
0
2,5
5
km
Legend
Main karst springs
Streams
Ponds
Springs
Railroads
Pasturelands test sites
Swallow hole
groundwater flow paths
Rižana protection zones
category 1
category 2
category 3
